# Platinum-Catalyzed Hydrosilylation in Polymer Chemistry

**DOI:** 10.3390/polym12102174

**Published:** 2020-09-23

**Authors:** Ruslan Yu. Lukin, Aidar M. Kuchkaev, Aleksandr V. Sukhov, Giyjaz E. Bekmukhamedov, Dmitry G. Yakhvarov

**Affiliations:** 1 Alexander Butlerov Institute of Chemistry, Kazan Federal University, 420008 Kazan, Russia; kuchkaev95@mail.ru (A.M.K.); alex.suhoff@rambler.ru (A.V.S.); gbekmouk@kpfu.ru (G.E.B.); 2 Arbuzov Institute of Organic and Physical Chemistry, FRC Kazan Scientific Center of the Russian Academy of Sciences, 420088 Kazan, Russia

**Keywords:** silicone polymers, hydrosilylation, organometallic catalysts, platinum complexes

## Abstract

This paper addresses a review of platinum-based hydrosilylation catalysts. The main field of application of these catalysts is the curing of silicone polymers. Since the 1960s, this area has developed rapidly in connection with the emergence of new polymer compositions and new areas of application. Here we describe general mechanisms of the catalyst activity and the structural effects of the ligands on activity and stability of the catalysts together with the methods for their synthesis.

## 1. Introduction

**Figure 1:**
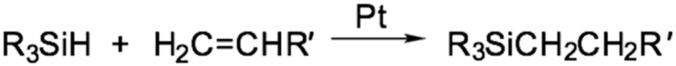
The common scheme of the platinum-catalyzed hydrosilylation reaction.

The hydrosilylation reaction (also referred to as hydrosilation) is widely used in the organosilicon industry. This reaction represents the addition of silicon-hydrogen bonds (Si–H) via an unsaturated carbon–carbon double bond (C=C), carbon–oxygen, and carbon–nitrogen double bonds. Hydrosilanes (as a general point, compounds containing Si–H bonds are more accurately called hydridosilanes (versus hydrosilanes) since the dipole moment of these bonds is pointing toward the hydrogen atom) are fairly inert concerning unsaturated compounds. The reaction is possible only under UV exposure, high temperature, or the presence of a catalyst. The general scheme of the hydrosilylation reaction is shown in Figure 1.

A huge number of catalysts involved in the hydrosilylation reactions and the catalysts based on transition metals of the platinum group are particularly effective [1,2]. In particular, platinum-based catalysts such as the Speier’s catalyst H_2_[PtCl_6_] and the Karstedt’s catalyst [Pt_2_(dvtms)_3_] (where “dvtms” is 1,3-divinyl-1,1,3,3-tetramethyldisiloxane) give a high number of catalytic cycles (TON), but at the same time display low selectivity. The solution to this problem was the use of bulky trialkylphosphine ligands, and later the use of platinum complexes with N-heterocyclic carbenes. These sterically hindered catalysts can increase stability and TON of catalysts of hydrosilylation reactions by making kinetic barriers for agglomeration of Pt(0) species. Steric effects to each catalytic steps are also important. The development of the [(t-Bu)_3_PPt(dvtms)] catalyst, which demonstrates high activity with a high level of requirements, also should be mentioned. The disadvantages of these catalytic systems are the high cost of Pt(0) precatalysts and the pyrophoric nature of the phosphines. The further rational development of catalysts rely on specific steric effects of ligands together with their electronic effects to central platinum atom, which is discussed below. 

## 2. Overview of Hydrosilylation

### 2.1. The General Mechanism of Action of Organometallic Catalysts in Hydrosilylation Processes

**Figure 2:**
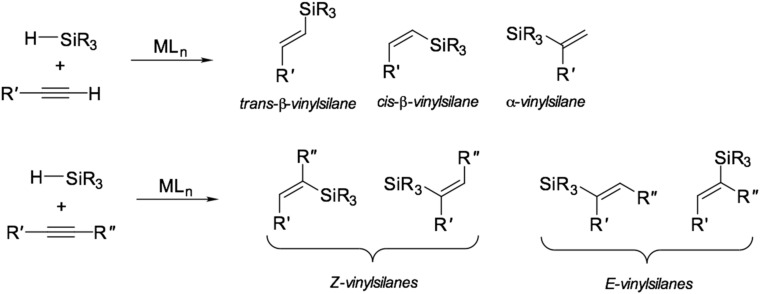
Common products of hydrosilylation of alkynes.

Hydrosilylation reaction is characterized by a wide range of substrates (compounds with multiple bonds) and reagents (silanes). The ability to control the yield of a particular isomer during the reaction by affecting the structure of the catalyst is one of the features of this reaction that distinguishes it from other processes in homogeneous organometallic catalysis. The general scheme of the hydrosilylation of alkynes is shown in Figure 2.

**Figure 3:**
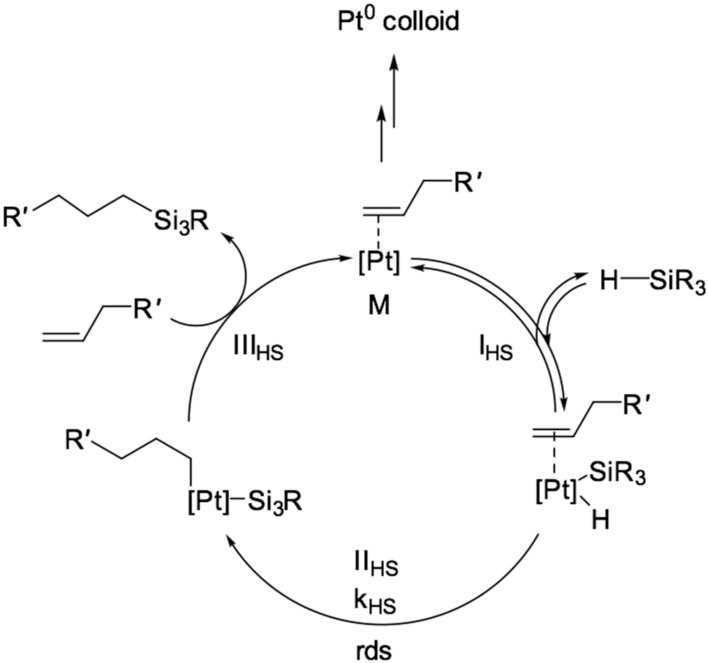
The Chalk-Harrod mechanism of hydrosilylation reaction of alkenes [3].

The platinum-catalyzed hydrosilylation reaction has been studied since the 1960s with the first reported work by Chalk and Harrod [3]. Following their work, modified mechanisms were proposed that explain the regioselectivity of the reaction not only for alkenes but also for alkynes [1,4,5]. Mechanisms have also been proposed for cases catalyzed by a wide range of other transition metal compounds. The classical mechanism, proposed by Chalk and Harrod in the 1960s, includes the reaction of the formation of the active form of the catalyst. It represents a complex of platinum with an alkenyl functional group, which undergoes the following transformations in the catalytic cycle:

I_HS_—oxidative addition of Si–H bonds to platinum;
II_HS_—migratory insertion of the coordinated alkene to Pt–H bond;
III_HS_—reductive elimination with the formation of Si–C bonds;
The mechanism of this transformation is presented in Figure 3.

**Figure 4:**
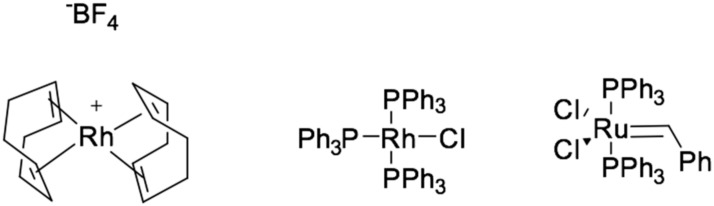
Examples of non-platinum hydrosilylation catalysts.

Other transition metal complexes also can be used as catalysts, such as the hydrosilylation reaction with complexes of rhodium [6,7], ruthenium [8,9,10], palladium [11,12], nickel [13], iron [14], iridium [15,16], examples are presented in Figure 4. There are cases of the reaction proceeding according to the modified Chalk and Harrod mechanism [17], in which the reaction rate is determined by the introduction of alkenes through M–Si bonds. An examples is hydrosilylation catalyzed by [Rh(PR3)3Cl], and calculations using density functional theory (DFT) have shown that the step of introducing ethylene into Rh–Si bonds is energetically more favorable than through Rh–H bonds.

Hydrosilylation of alkenes catalyzed by transition metal complexes is often accompanied by side reactions such as isomerization, polymerization, oligomerization, and hydrogenation [18]. One of the most common side reactions is dehydrogenative silylation, which takes place in the presence of an intermediate of alkene insertion into M–Si bond, which is most common for complexes of the iron and cobalt group [19]. In paper [20], the mechanism of Pt(0)-catalyzed hydrosilylation of alkenes was studied using ^2^H-labeling. It was found that the Karstedt’s system catalyzes the isomerization reaction of terminal alkenes. In the same work, the reversible nature of the alkene incorporation stage by the Pt–H bond was studied.

An important process occurring in the catalytic system in the case of using platinum(0) complexes is the formation of platinum black. The mechanism of its formation and effect on catalytic activity was described in [21]. It was shown that platinum in colloidal form does not catalyze this reaction. Previous studies reported that formation of platinum colloids was a key step in hydrosilylation. It is now clear that colloid formation occurs as an end stage of the reaction. X-ray absorption fine structure (EXAFS) analysis has shown that molecular compounds are present during hydrosilylation. Colloidal platinum is observed by transmission electron microscopy (TEM) after evaporation of solutions from several reactions that involve platinum, a Si–H compound, and either poorly coordinating olefins or no olefin. However, in some cases where silicon-vinyl-containing species were present, the reaction product between platinum and a Si–H-containing compound did not give colloidal platinum species; and TEM analysis showed that the crystalline material present was not metallic platinum crystallites [21].

Among commercially used catalysts, the most common complexes are based on Pt(0) because of their high activity at relatively low concentrations in the hydrosilylation process. The main commercially available catalysts are the Karstedt’s, Speier’s, Ashby’s, and Lamoreaux catalysts together with a series of non-platinum containing catalysts [22]. The main advantages of the latter systems compared to other platinum compounds are: easy modifiability by various inhibitors and stabilizing ligands and simplicity of synthesis. 

### 2.2. Hydrosilylation in the Crosslinking of Polydimethylsiloxane Polymers

The hydrosilylation crosslinking of silicone rubber has been increasingly used in rubber manufacture because of many technological and economic advantages. The composition and proportion of both polysiloxane components as well as the catalyst used (mostly Pt-complex-based), determine the quality of the vulcanisate and in particular allow obtaining materials of given properties and for special applications. Among the recent patents, some describe procedures for preparation of silicon rubber excellently improving its chemical and temperature resistance mechanical properties such as tensile strength and high tear strength gas permeability and other desirable properties of gels. In particular, fluorinated curable silicone composition cures into a gel with improved chemical and solvent resistance. They constitute protective coating of such commonly used materials as optical displays, textiles, metals, stone, wood, and leather [1]. It was possible to print drug-free and drug-loaded structures of polydimethylsiloxane (PDMS) with the 3D printing technique with the use of photoactive platinum catalysts [23]. Light post-curing was a suitable technique to crosslink prednisolone containing PDMS structures.

The rheology of the systems can also vary widely, ranging from dip-cures to liquid injection molding (LIM) and conventional heat cure rubber (HCR) processing. Vinyl terminated polydimethylsiloxane polymers (viscosity > 200 cSt) are typically crosslinked by methylhydrosiloxane–dimethylsiloxane copolymer with 15–50 mole % of polymethylhydrosiloxane. The catalyst is usually a complex of platinum in alcohol, xylene, divinylsiloxanes, or cyclic vinylsiloxanes. The system is mostly prepared in two parts (part A—vinylsiloxane + Pt (5–10 ppm), part B—hydrosilox- ane + inhibitor, generally 0.01–50 ppm). Apart from temperature, also moderators and inhibitors are used to control the work time. Moderators (e.g., tetravinyltetramethylcyclotetrasiloxane) slow Pt catalysis. Inhibitors stop the platinum catalyst, they are volatile or are decomposed by heat or light (UV) [1]. 

This reaction is indeed strongly exothermic, more so than almost any other polymerization, but the exotherm and the reaction rate can be controlled easily by adjusting the platinum concentration and reaction temperature. Authors in [24] demonstrated the preparation of extremely cross-linked poly(dimethylsiloxane) (PDMS)-based material D_4_H/D_4_V and reported optical, mechanical, and surface properties. To prepare the samples authors used a very low catalyst concentration (12 ppm Pt) and a two-stage cure at different temperatures. For certain applications (e.g., reaction injection molding), an exotherm and rapid reaction may be desirable, and these could be tuned with the catalyst concentration. Transparent monolithic molded objects are prepared catalytically with no byproducts; parts per million levels of platinum (catalyst) remain in the articles. Essentially the same material was prepared in 1993 and described as a “hard transparent glass.” The catalytic reaction used was reported in 1999 always to exhibit a “violent exotherm,” can be controlled conveniently using a low (parts per million) catalyst concentration. Authors in [24] showed the combination of low surface energy, transparency, hardness, elasticity, and thermal stability makes this an unusual and interesting material. Reported are demonstrations that D_4_H/D_4_V silicone (the product of the platinum-catalyzed hydrosilylation reaction between tetramethylcyclotetrasiloxane and tetramethyltetravinylcyclotetrasiloxane) is useful and practical as a replica material for both nanoimprint lithography (NIL) and capillary force lithography (CFL). The multiple advantageous properties of this extremely cross-linked material include UV transparency (for photo NIL and photo CFL), thermal stability (for high printing temperatures), high modulus (for high printing pressures), low surface energy (for easy demolding), and low viscosity precursors (for replicating small scale features).

### 2.3. Karstedt’s Catalyst

**Figure 5:**
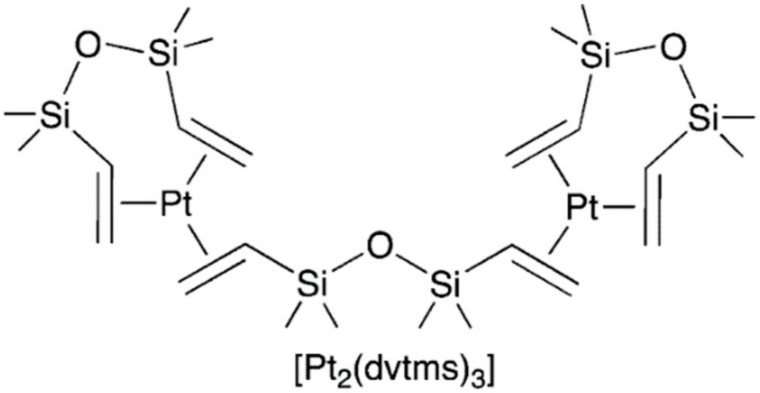
The structure of binuclear platinum(0) complex with 1,3-divinyl-1,1,3,3-tetramethyldisiloxane (dvtms).

This catalyst is one of the most common in the silicone industry and is a solution of platinum complexes in the zero oxidation state, with a platinum concentration of 2.5–5.0 wt%, calculated as Pt in 1,3-divinyl-1,1,3,3-tetramethyldisiloxane dvtms. The general formula of this complex is: [Ptx(dvtms)y]. The observed stability of the composition is due to the fact that the labile ligand dvtms is in excess for platinum, thereby stabilizing it, forming an equilibrium mixture. In the 1960s, Dow Corning [25] and Karstedt (General Electric) [26] studied the reaction of hexachloroplatinic acid with vinyl-containing siloxanes. As a result, the formation of platinum-soluble complexes in silicone (0) was observed, when trying to isolate which the platinum black was formed. The instability of these compounds in their pure form for a long time did not allow us to determine the data structure. Lappert et al. studied the reaction of dicyclooctadienylplatinum with dvtms and discovered the formation of a binuclear complex of the general formula [Pt2(dvtms)3]. The formula of this complex is shown in Figure 5.

**Figure 6:**
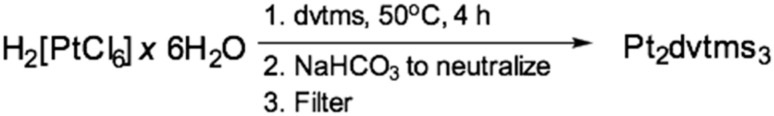
The scheme of Karstedt’s catalyst preparation.

The literature describes many methods for producing similar forms of catalysts. Thus, in the original patent [26] a method for preparation diethylene diplatin from platinum tetrachloride is described. For the catalytic test 19.5 parts of [Pt(C2H4)Cl2]2 were added to 27.8 parts of 1,3-divinyl-1,1,3,3-tetramethyldisiloxane, after which the mixture was kept under stirring for 1 h at 30 °C, then benzene was added to the resulting mixture and left to cool for 2 h. Subsequently, 48 parts of ethyl alcohol and sodium bicarbonate were added to the solution until the exothermic reaction ceased and gas was released, after which the mixture was filtered and the filtrate was concentrated in vacuo. This complex can be similarly obtained from sodium tetrachloroplatinate by reaction with a two-fold excess of dvtms in ethanol. For this, 25 parts of tetrachloroplatinate, 125 parts of EtOH, 50 parts of dvtms, 25 parts of NaHCO3 are used. The resulting mixture is an oil that crystallizes at −13 °C. By nuclear magnetic resonance (NMR)-analysis it was shown that the structure of the complex, presented in Figure 5, dominates in the mixture. In the same patent, a method for producing a catalyst from platinum hydrochloric acid is indicated. To 10 parts of H2PtCl6×6H2O, 20 parts of sodium bicarbonate, 20 parts of dvtms, and 50 parts of ethanol is added. After heating under reflux for 30 min and keeping at room temperature for 13 h and concentration in vacuo, 17 parts of a liquid product were isolated, which were further purified from salt by dissolving in benzene. The scheme of Karstedt’s catalyst preparation is shown in Figure 6.

**Figure 7:**
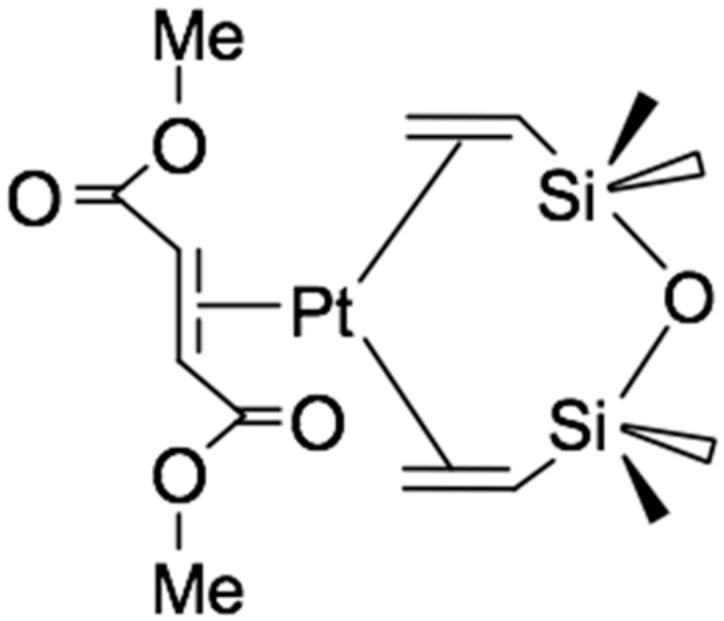
The structure of the complex formed by the reaction of dimethyl fumarate with a Karstedt’s catalyst.

Most often, alkynes and alkenes with electron-withdrawing substituents are used as inhibitors. Their effect is manifested in the formation of relatively inert complexes of platinum (0), preventing the occurrence of hydrosilylation reactions at room temperature during the period of molding or storage of the silicone mixture. The most commonly used inhibitors are maleates and fumarates [26,27]. Thus, in [21], the mechanism of action of dimethyl maleate and dimethyl fumarate as an inhibitor of the reaction of alkenes with silanes was studied. Using ^13^C NMR spectroscopy it was found that when 4 equivalents of dimethyl fumarate are added to the Karstedt’s catalyst, the complex shown in Figure 7 is formed. The formation of a similar complex is observed in the case of using 4 equivalents of dimethyl maleate.

In the same work, the properties of the obtained systems were studied in the process of silicone curing using differential scanning calorimetry. So, for all three systems (Karstedt’s catalyst + 35 equivalents of inhibitor), 2 peaks were observed, the first of which corresponded to the activation of the catalyst due to the hydrosilylation of the bound inhibitor. Second, the main peak corresponded to the process of curing of the silicone mixture. The most effective according to the estimated amount of heat released per 1 g of the mixture is a mixture containing dimethyl maleate as an inhibitor.

Catalytic systems based on various alkyl derivatives have also been studied. The most commonly used is 1-ethynylcyclohexanol. So, in the patent [28], a comparative study of 2-methyl-3-butyn-2-ol and 2,4,7,9-tetramethyl-5-decyne-4,7-diol was carried out. When using 1-ethynylcyclohexanol (ECH) in a 30-fold excess with respect to the platinum, the resulting mixture has a life of 24 h at 25 °C and 7 h at 40 °C. The beginning of the curing process according to differential scanning calorimetry (DSC) study comes at 99 °C. The use of a multiple-fold excess of 2,4,7,9-tetramethyl-5-decyne-4,7-diol increases the curing temperature up to 123 °C and increases the pot-life time of the cured mixture up to 48 h at 25 °C. The use of 2-methyl-2-pentyn-3-ol in the same proportions gives a mixture curable at 99 °C with the amount of heat released in a much larger amount than in the two previous cases, which indicates increased activity of the catalyst. 

In the same patent, the inhibitory properties of diallyl maleate and tetravinyl-tetramethyltetrasiloxane were studied by a similar method. In the case of diallyl maleate, curing began to proceed at 108 °C, while for tetravinyl-tetramethyltetrasiloxane this temperature was 73 °C.

**Figure 8:**
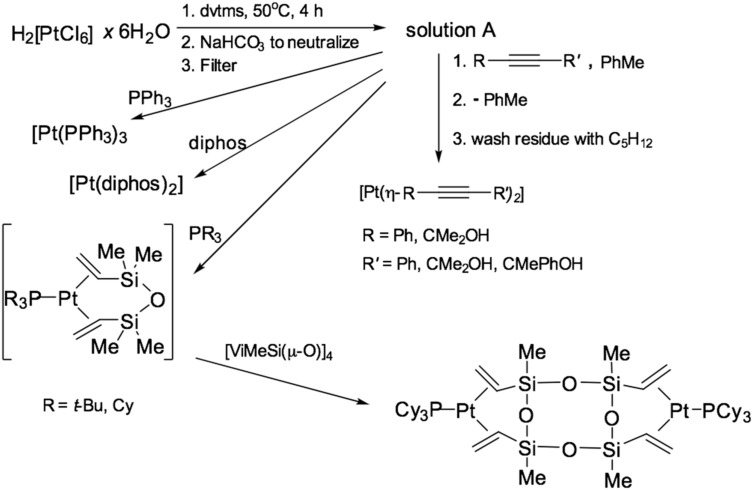
The scheme for the preparation of platinum (0) complexes with phosphines.

The patents [29,30,31] also describe the use of dialkyl esters of acetylenedicarboxylic acid. So when using dimethyl acetylenedicarboxylate (DMAD) in various proportions (from 25 to 100), the resulting mixture does not undergo curing at room temperature for a long period if a large excess of inhibitor is used. With a 500-fold by weight excess of DMAD, the relative change in viscosity when kept for 6 h is 3% and rises to 35% when the excess is reduced to 60-fold. A similar effect is manifested when using DMAD. Methods for preparing a rubber curing catalyst using a Karstedt’s catalyst as a precursor by directly adding a ligand are also described. Example [32] describes a method for producing a complex of platinum(0) with alkynes such as 4-methyl-1-pentyn-3-ol, 3-ethyl-1-pentyn-3-ol, as well as other derivatives of propargyl alcohols. The radicals used are shown in Figure 8.

Thus, in a typical example, to the mixture obtained by the reaction between platinum hydrochloric acid and dvtms, a solution of alkyne in toluene is added, after which the mixture is stirred and the product is isolated by evaporation of the solvent, followed by washing in pentane. The resulting complexes have the formula Pt(alkyne)_2_.

The use of combined inhibitory mixtures was also studied. For example, the patent [33] describes the use of an inhibitor obtained by mixing 500 parts of ethynylcyclohexanol and from 0.01 to 0.4 parts of tert-butyl hydroperoxide or cumyl hydroperoxide. The resulting mixture exhibits greater inhibitory ability than a single ethynylcyclohexanol. Examples of inhibition of Karstedt’s catalyst by various hydroperoxides are also described [4].

In the patent [34], derivatives of ethynylalkenes are used as inhibitors for the curing of silicone rubber for high voltage insulation. These are: 3-methyl-3-penten-1-yne, 3-methyl-3-hexen-1-yne, 3,5- dimethyl-3-hexen-1-yne, 3-ethyl-3-buten-1-yne, 3-phenyl-3-buten-1-yne. The ratio of catalyst: inhibitor ranged from 1:1 to 1:100, the ratio of 1:50 being most preferred. The resulting catalyst was tested on a mixture prepared based on polydimethylsiloxane terminated with vinyl groups with a viscosity of about 10,000 mPa⋅s. These platinum catalysts were also used for the preparation of mixtures based on high molecular weight rubbers containing about 0.13 mol% of vinyl groups.

In a similar way, systems are used that have 2,2′-bipyridyl (bpy) ligands as an inhibitor [35], which is added to the Karstedt’s catalyst in 15-fold excess. As a result of the reaction, a complex of platinum with 1,3-divinyl-1,1,3,3-tetramethyldisiloxane and bipyridyl was obtained in the isolation of compound [Pt(dvtms)(bpy)]. The addition of this inhibitor significantly increases the pot-life time of the composition. For example, in all cases, an excess of 2,2′-bipyridyl is used as an inhibitor; the change in viscosity does not exceed 18% of the initial one. Also, platinum(0) complexes with N,N-chelate heterocyclic ligands were studied in [36]. The effect of multifunctional co-ligands on catalysis by platinum complexes, in particular, Karstedt’s catalyst, was studied in [37]. A number Aryl-BINMOL (1,1′-binaphthalene-2-α-arylmethanol-2′-ols) type diols were used as ligands. These ligands possess chirality and can coordinate with the central atom of platinum in several modes (C=C–bond, N, P, O). When using a Karstedt’s catalyst combined with these ligands in a 1: 2 molar ratio, the phenylacetylene hydrosilylation reaction showed a change in the selectivity of the reaction toward an increase in the yield of α-vinyl silanes, compared with a system without co-ligands, while the β-isomer remains the dominant product in all cases.

### 2.4. Platinum Phosphine Complexes

Methods for the preparation of vinyl siloxane complexes of Pt(0) with phosphines were published in [38,39]. These complexes were patented as curing catalysts [40]. Also in these works, the concept of obtaining complexes of platinum(0) was described based on a solution containing vinylsiloxane–platinum (solution A), similar to the Karstedt’s catalyst. Methods for the preparation of complexes of the type [Pt(PR3)3] and [Pt(dvtms)(PR3)] were developed, and it was also found that platinum in this form was suitable for the preparation of its complexes with alkynes, which made it possible to replace expensive reagents of the [Pt(COD)2] and [Pt(nbe)3] (nbe—norbornene) to the equilibrium mixture A. The scheme for the preparation of platinum (0) complexes based on the Karstedt’s catalyst is shown in Figure 8.

**Figure 9:**
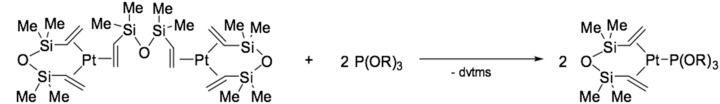
Reaction of Karstedt’s catalyst with triorganophosphites.

The catalytic properties of the complexes obtained by the reaction between Karstedt’s catalyst and a series of triorganophosphites (Figure 9) were studied in [41,42].

The resulting mixtures had a longer pot-life time at room temperature and greater stability of the platinum catalyst. High curing rate at elevated temperatures (from 90 to 130 °C) was noted. From the structural correlation, it was found that complexes containing sterically bulky phosphites are most suitable for use in the curing of silicone mixtures. Also, it is worth noting the stability of these catalysts to decomposition and loss of catalytic activity

[Pt{h^4^-(H_2_C=CHSiMe_2_)_2_O}{P(O(2-*tert*-Bu-4-MeC_6_H_3_))_3_}] was studied using ^31^P NMR spectroscopy. It was found that during the storage of this compound in a liquid silicone mixture, only 1.47% of the catalyst underwent decomposition.

In the patent [43], the use of cyclic organosiloxanes of the general formula (Me(CH_2_=CH)SiO)_n_ as inhibitors was proposed. The composition of the inhibitory mixture is as follows: 528 parts of 2,4,6-trimethyl-2,4,6-tris-3,3,3-trifluoropropylcyclo-trisiloxane, 83 parts of 2,4,6-trimethyl-2,4,6 -trivitinylcyclotrisiloxane, 27 parts of vinyl-terminated dimethylsiloxane oligomer containing an average of 6 dimethylsiloxane units per formula. The resulting mixture prevented curing up to 96 h. 

The patent [44] provides a series of catalysts based on platinum(0) complexes with phosphines and trialkyl phosphites. Examples are: [(CH_3_)_3_P]_4_Pt, [(C_4_H_9_)_3_P]_4_Pt, [(C_6_H_5_)_3_P]_4_Pt, [(CH_3_O)_3_P]_4_Pt, [(C_6_H_5_O)_3_P]_4_Pt, [(C_4_H_9_O)_3_P]_4_Pt, [(*p*-ClC_6_H_4_O)_3_P]_4_Pt, [(C_2_H_5_)_2_(C_6_H_5_)P]_4_Pt, (C_6_H_5_)_3_P[(*p*-ClC_6_H_4_O)_3_P]_3_Pt, [(C_2_H_5_O)_2_(C_6_H_5_O)P]_4_Pt. The test of the above catalysts was carried out on a mixture obtained from 100 parts of polyorganosiloxane containing 0.2 mol% of methylvinylsiloxane fragments with a polymerization degree of about 6000, 40 parts of aerosil, 2 parts of polyorganosiloxane diol with a viscosity of 50 cSt and 0.5 parts of polymethylhydrosiloxane. To this mixture a catalyst in an amount of 0.01 parts by weight was added. The resulting mixture had a longer lifespan compared to cases where a Speier’s catalyst was used. Silicone additive curing compositions using platinum complexes bearing tris(2,4-di-tert-butylphenyl)phosphite were also developed [45]. The example describes the use of vinyl terminated polydimethylsiloxane with a concentration of vinyl groups of 0.2 mmol/g (883 parts by weight), 13 parts of a copolymer of dimethyl- and methylhydrosiloxane containing Si-H groups of 7 mmol/g. The use of excess tris(2,4-di-tert-butylphenyl) phosphite led to an increase in the lifetime up to 6 months. The curing temperature was 120 °C. Authors in [46] described the use of Buchwald-type co-ligands in the hydrosilylation of Vi-PDMS and H-PDMS with the Karstedt’s catalyst allows one to sufficiently increase the pot-life of polymeric silicone compositions. Application of these ligands provides high stability of thus formed catalysts at high concentrations depriving formation of platinum colloids. This can be explained by specific steric and electronic effects of coordinated Buchwald-type ligands on the rate-determining step of the alkene insertion into the formed intermediate Pt–H bond

### 2.5. Platinum(0) Complexes with Carbenes

**Figure 10:**
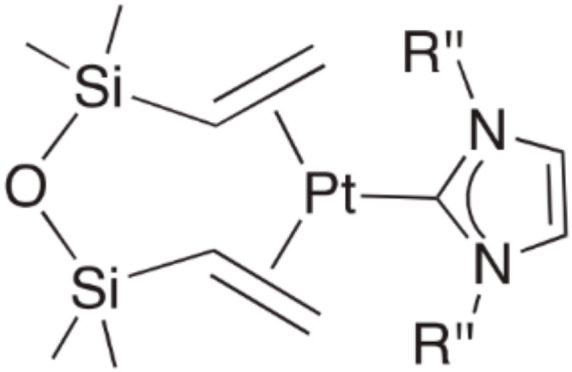
General structure of platinum carbene complexes with dvtms.

These complexes were thoroughly studied in [47,48,49,50,51]; in the case of N-heterocyclic carbene (NHC) complexes, the Karstedt’s catalyst solution was used as the source of platinum(0). The general structure of the first generation catalyst is shown in Figure 10 (R’’—alkyl- or aryl- substituents).

**Figure 11:**
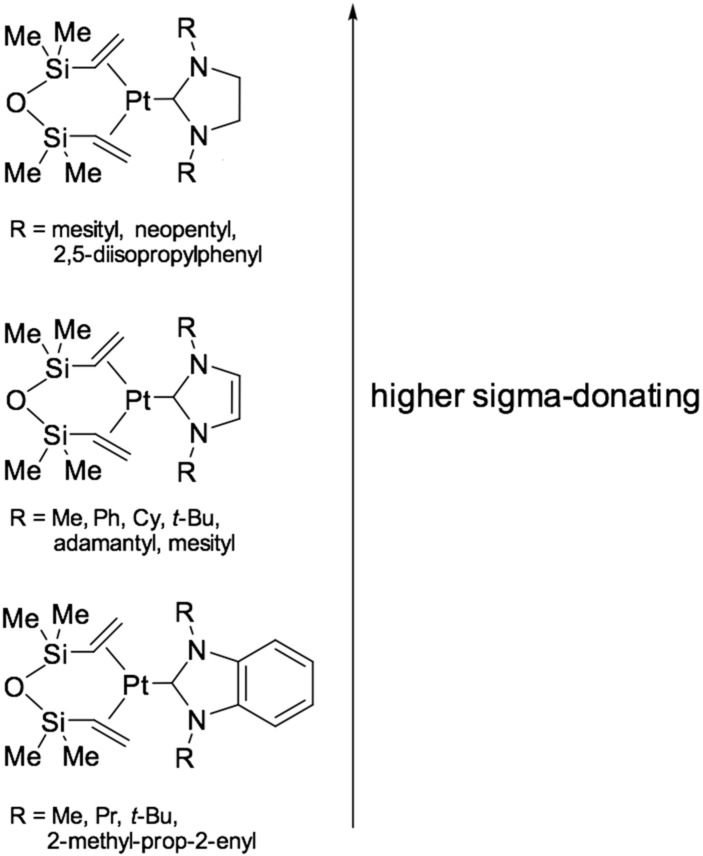
Three generations of platinum carbene complexes.

These catalysts were developed to stabilize Pt-intermediates by high strength of Pt-carbene bond from aggregation and platinum colloid formation. In a typical experiment for the preparation of carbene [47] platinum complexes with dvtms, a Karstedt’s catalyst solution in toluene is used, to which a two-fold excess of imidazole salt and potassium tert-butylate are added as the base for deprotonation of the C-2 atom of the imidazole salt. After that, the solution is filtered off and concentrated in vacuo to a white precipitate. Next, the precipitate is washed with tetrahydrofuran and recrystallized from isopropanol. In the case of using triazole salts, it is also possible to use sodium acetate as a base. The resulting catalysts are highly resistant to degradation. Studies using UV-spectroscopy [47] have not shown the presence of colloidal particles of platinum(0). Figure 11 illustrates the 1st and 2nd generation of catalysts based on platinum(0) complexes with dvtms and N-heterocyclic carbenes.

**Figure 12:**
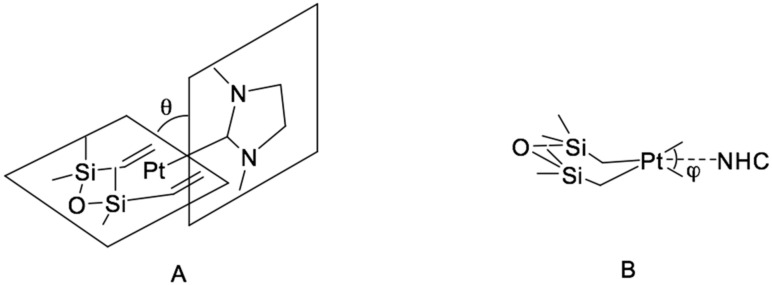
Projections of [(NHC)Pt(dvtms)] structure in terms of the tilt angle (**A**) and the torsion angle (**B**).

The resulting catalysts exhibit high efficiency in the hydrosilylation of 1-octene with heptamethyltrisiloxane, showing a higher degree of conversion compared to the classic Karstedt’s catalyst. This catalyst also possessed high selectivity in the hydrosilylation of 4-vinylcyclohexene oxide with a copolymer of methylhydrosiloxane and dimethylsiloxane of the formula Me_3_SiO-(Me_2_SiO)_85_-(MeHSiO)_7_-SiMe_3_, with a conversion rate of 95% while the Karstedt’s catalyst gave a conversion of not more than 50% because of the adverse reactions with the epoxy fragment when using platinum in an amount of 10 ppm. Using X-ray diffraction [47,48,50], the structure of these complexes was determined (Figure 12). The main geometric parameters characterizing the above compounds are the tilt angle θ and the torsion angle φ, which reflects the volume of the carbene substituent.

**Figure 13:**
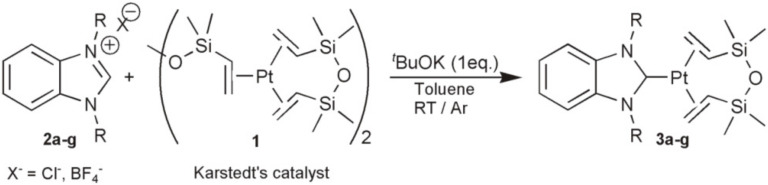
The synthesis of platinum(0) catalysts using benzoimidazole derivatives.

A method for the synthesis of platinum(0) catalysts using benzoimidazole derivatives (Figure 13) is also given in [51]. These complexes are more active because of an increase in the sigma-donating ability of the carbene ligand.

A number of hydrosilylation catalysts based on platinum(0) complexes with dvtms and imidazolo[1,5-a]pyridin-3-ylidene were published in [47], the authors carried out a directed design of these complexes since the aforementioned carbene ligand has the most pronounced sigma-donating ability compared to other carbenes. The authors also studied the correlation of catalytic activity (reaction rate) and the difference in the energy of LUMO/HOMO (Highest Occupied Molecular Orbital/Lowest Unoccupied Molecular Orbital). More sterically hindered carbene ligands were introduced in later work [52]. Examples are: [Pt(IPr*)(dvtms)] (where, IPr* = 1,3-bis{2,6-bis(diphenylmethyl)-4-methylphenyl}imidazol-2-ylidene) and [Pt(IPr*OMe)(dvtms)] (where, IPr*OMe = 1,3-bis{2,6-bis(diphenylmethyl)-4-methoxyphenyl}imidazol-2-ylidene). It was demonstrated that the postulated dependence of selectivities of hydrosilylation processes on the steric bulk of the NHC ligand remains true for complexes containing N-heterocyclic carbene ligands significantly bulkier (Nolan height parameter AH > 200°) than the previously described complexes. The complexes efficiently catalyze the highly regio- and chemoselective hydrosilylation of a wide range of functionalized terminal olefins, as well as terminal and internal acetylenes. Moreover, the complexes tested are characterized by particularly high values of TON, which reach 107 for the hydrosilylation of olefins.

**Figure 14:**
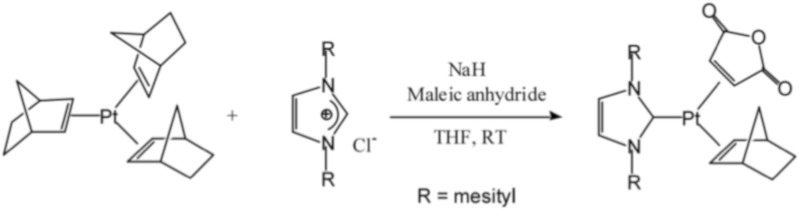
Formation of carbene platinum complexes with maleic anhydride.

In [53], a series of catalysts were presented, which are complexes of platinum(0) with carbene and two dialkylfumarate molecules. These complexes were obtained using Pt(norbornene)3 as well as imidazole salt and maleic anhydride in tetrahydrofuran, as shown in Figure 14.

### 2.6. Lamoreaux Catalyst

This type of catalyst is obtained by the reduction of hexachloroplatinum acid with alcohols containing long-chained substituents which are also used to increase the solubility of the catalyst in non-polar organic solvents such as benzene, toluene, xylene, and hexane [54,55].

The general method of preparation of Lamoreaux catalyst is as follows. Hexachloroplatinum acid hydrate is dissolved in an excess amount of the corresponding alcohol, the mixture is heated at 70–75 °C. The obtained water and hydrogen chloride are removed under reduced pressure. The final form of the catalyst is a red-brown liquid. There are examples of such catalyst obtained using octyl, isoamyl, 2-ethylhexyl, hexyl, and pentyl alcohols. In all cases, the by-products were aldehydes and ethers.

This type of catalyst was tested in the hydrosilylation reaction of vinyl acetate with methyldichlorosilane, as well as in the processes of crosslinking a silicone mixture containing methyl vinyl fragments with a copolymer of methylhydrosiloxane and dimethylsiloxane. Lamoreaux catalyst exhibits greater stability compared to Karstedt’s catalyst, however, it is active even at room temperature which restricts its industrial applications.

### 2.7. Ashby’s Catalyst

**Figure 15:**
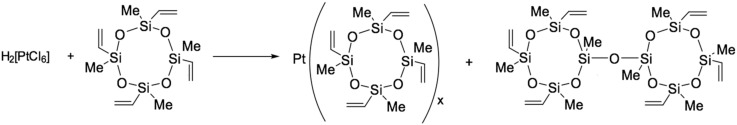
Preparation of Ashby’s catalysts.

There is a number of works describing the methods of synthesis of Pt(0) complexes with 1,3,5,7-tetramethyl-1,3,5,7-tetravinylcyclotetrasiloxane [56,57]. The use of tetradentate ligand increases stability of platinum complexes by strong chelate effect. A general method of producing this type of catalyst is similar to the preparation of Karstedt’s catalyst; it is based on the reaction of dichlorodiethylene platinum or hexachloroplatinic acid with cyclic methylvinylsiloxane, as it is shown in Figure 15.

### 2.8. Catalysts Based on Pt(II) Complexes

The patent [58] describes a method for producing a catalyst based on platinum(II) complexes with styrene. A general concept of obtaining these compounds consists in the reaction of halogen-containing platinum compounds with styrene in the presence of bases and reducing agents. Polar compounds, such as alcohols, as well as aromatic hydrocarbons, are used as a solvent for these reactions.

In the above example, an orange crystalline compound with Pt:Cl ratio of 1:3 is obtained, which corresponds to the formation of Pt(II) complex of the composition Na[Pt(PhCH=CH_2_)Cl_3_]. It is noted that heating the complex leads to a change in its composition. With an increase in temperature and heating time the Pt:Cl ratio decreases from 1:4 to 1:0.9. The catalyst was made by dissolving the obtained platinum complexes to a platinum concentration in solution equal to 0.75%. The tests were carried out on a two-component silicone mixture with the catalyst content of 1.6 parts per 200 parts of polydimethylsiloxane. The resulting mixture exhibited a curing period of 40–50 s in the case of a platinum complex with styrene and the Pt:Cl ratio of 1:3.1 or 1:2.8. It was found that the activity of the catalyst significantly decreases both with an increase of chlorine content (formation of [PtCl_4_]^2−^ complexes) and with its decrease (formation of inactive binuclear complexes, such as [Pt(PhCH=CH_2_)Cl_2_]_2_ and formation of Pt(0) complexes).

**Figure 16:**
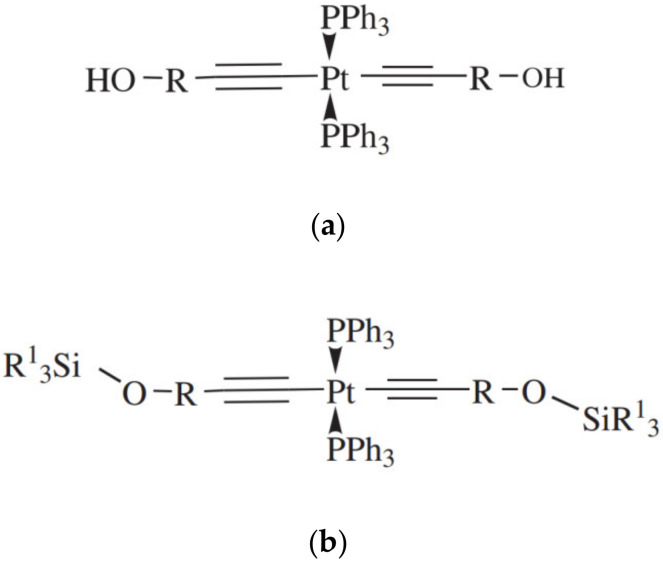
Structure of the catalysts based on Pt complexes with diacetylenyl ligands (**a**) R—1-cyclohexyl, isopropyl; (**b**) R^1^—CH_3_, CH_3_CH_2_, PhCH_2_CH_2_).

Platinum complexes containing triphenylphosphine and monosubstituted acetylides were investigated in [59]. The general formula of the compounds is shown in Figure 16.

Mentioned complexes are obtained by adding a small excess of alkyne to trans-[Pt(Ph_3_P)_2_Cl_2_)] in the presence of CuI and base. The catalysts were tested on a silicone composition with [Si–H]/[Si–CH=CH_2_] ratio of 1.2 and the catalyst concentration of 8 ppm. In all cases, a relatively long lifetime of the finished mixtures (from 3 to 15 days) along with rapid curing at elevated temperatures were observed. 

### 2.9. Encapsulated Catalysts

Several catalysts with a form of usage, which is different from all of the above considered, have also been proposed. This method consists of microencapsulation of the catalyst, which then can be used for one-component silicone mixtures of hot curing [55].

Such catalytic systems can be obtained by the reaction between [(COD)PtCl_2_] and cyclodextrines (preferably β-CD; CD = cyclodextrin). In this case the supramolecular host-guest type complexes [Pt(COD)(CD)Cl_2_] are formed [60,61]. It is possible to reversibly remove the [(COD)PtCl_2_] from these complexes by leaching [62]. The resulting platinum catalyst is highly stable in silicone mixtures. Thus, lifetimes exceeding one month have been achieved for one-part hot curing silicone mixtures, while curing at 200 °C takes 85 s. However, a limiting factor in the use of such catalysts is their low solubility in siloxane polymers, which leads to non-uniform curing. These complexes can be immobilized on the silica gel, containing surface-grafted organic groups. The resulting system can be easily incorporated into long-life silicone mixtures [62].

Another approach is to incorporate a hydrosilylation catalyst into thermoplastic polymers or into crystalline compounds, which melt at elevated temperatures. Such introduction can be performed using monomeric organic compounds (3,6-dimethyl-4-octyne-3,6-diol, melting point mp = 54 °C; thiochroman-4-ol, mp = 69 °C; 2-acetyl-1-tetralone, mp =56 °C). But in most cases, the particles of thermoplastic organopolysiloxane rubbers are used as a matrix for incorporation. This matrix melts at the temperature of 40–200 °C, and thus releases a catalyst. The possibility of varying material and the size of thermoplastic particles allows to control the lifetime, as well as the curing temperature of the resulting rubber compounds. Also, these systems can be modified by inhibitors [63] and stabilizing disiloxanes [64]. These mixtures can be optimized taking into account the glass transition temperature and the particle size. The optimum glass transition temperature should be in the range of 40–250 °C, and the particle size should be 0.1–20.0 micrometers [65].

### 2.10. “Sleeping” Platinum Complexes with Inhibitory Ligands

This concept implies the use of platinum complexes with a strong platinum–ligand bond. These complexes are catalytically inactive at room temperature, but provide a high hydrosilylation rate at elevated temperature. For example, platinum(II) complexes [R_2_Pt[(P(OR)_3_]_2_] (R = halogen, alkoxyl, alkyl) have relatively low activation temperatures (100–160 °C), and also prevent the formation of colloidal platinum particles, which in turn increases the catalyst lifetime and eliminates the undesirable coloring of the final mixture [66]. The platinum bisalkynyl complexes [(COD)Pt(C≡CR)_2_] and [(bicyclo[2,2,1]-hepta-2,5-diene)Pt(C≡CR)_2_] possess similar catalytic properties [67]. These complexes are highly soluble in polysiloxanes, inactive up to 40 °C, and do not decompose with the formation of colloidal platinum particles. A number of such catalysts were successfully tested in the hydrosilylation reaction to obtain a silicone coating of papers and fabrics [68]. Using DFT quantum chemical calculations it was shown that the activation of these complexes is initiated by the oxidative addition of the Si–H bond. The activation energy and the activation temperature significantly depend on the nature of the substituent in the alkynyl group [69].

**Figure 17:**
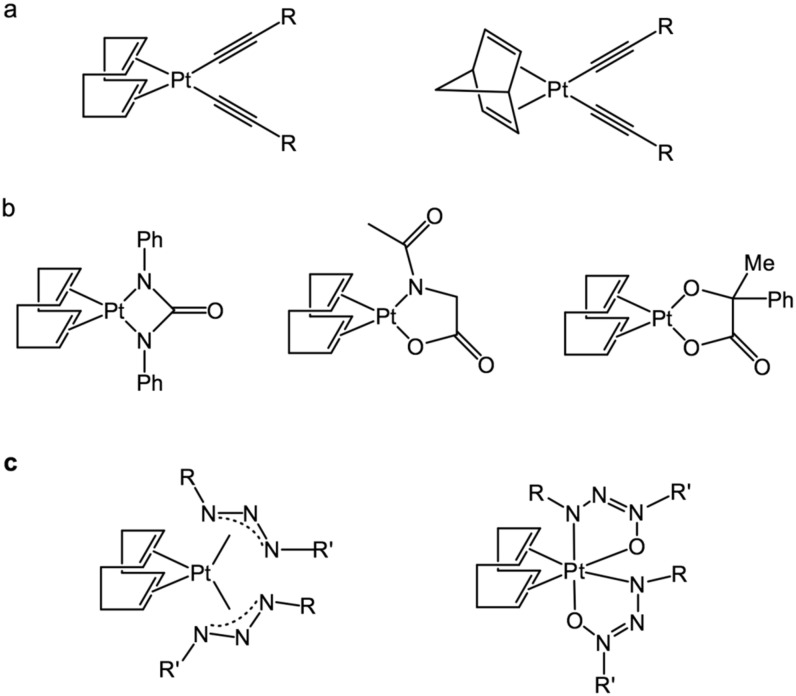
“Sleeping” platinum catalysts are based on (**a**) bisalkynes; (**b**) carbamides and bidendate acids, triazenes; (**c**) triazenoxides.

Other examples of the “sleeping” platinum complexes are the compounds containing bidentate ligands based on urea moiety (*N*,*N*-diphenylcarbamide; *N*-(n-hexyl)-*N*-phenylcarbamide; *N*,*N*-diacetylcarbamide), acidic moiety (phenylphosphinic, *N*-acetylglycine, mandelic acid), as well as on phenyl-isocyanate. The listed complexes have relatively low activation temperature, exhibit the high catalytic performance of the activated state, and they exclude the problem of colloidal platinum formation [70]. Examples of “sleeping” platinum catalysts, based on bisalkynes, carbamides, bidendate acids, triazenes, and triazenoxides, are shown in Figure 17. 

Platinum complexes bearing triazenes and their derivatives—[(Pt(PhNNN(CH_2_)_5_Me)_4_)], [(COD)Pt((p-NO_2_-Ph)NNN(CH2)_7_Me)_2_)]—have high activation energies at room temperature, and therefore prevent the hydrosilylation reaction at this conditions, even in the absence of an inhibitor [71]. Further development of this direction led to platinum complexes based on triazenoxides. The activation energy of these complexes can be modulated by varying the ligand structure. These catalytic systems can be activated by heating to a temperature of 50–250 °C, as well as by the irradiation with UV-light. According to patent [72], these complexes are obtained by mixing two equivalents of triazene and excessive amounts of the base, followed by the addition of one equivalent of [Pt(COD)Cl_2_], dissolved in ethanol, after that the resulting complex is isolated as a precipitate.

### 2.11. Photoactivated Hydrosilylation

Photogenerated catalysts obtained by irradiation with UV light are also of particular interest to researchers in the field of hydrosilylation [72]. They combine such advantages as high stability of the resulting mixtures at room temperature and rapid curing under exposure to UV rays, which is especially important for rubber compounds containing thermolabile components and for low viscosity composites. However, the main disadvantage of photoactivated catalysts is the non-uniform curing in the case of using thick rubber layers, which is due to the difference in optical density at different points.

Generally, photoactivated catalytic systems are obtained by combining platinum complexes with inhibitors (alkynols, alkenes, phosphines, etc.,) and photosensitizers (benzophenone, acetophenone). The latter are usually excited upon irradiation and then transfer energy to a catalyst, thus activating it [73]. As mentioned above, platinum complexes with triazenes and their derivatives are just as sensitive to light as they are to high temperatures. The photolytic decomposition of [Pt(PhNNN(CH2)5Me)4] upon irradiation with XeCl* excimers obtained with a laser (wavelength 308 nm) was studied by the authors of [74]. As a result of this exposure, coordinatively unsaturated platinum complexes are generated that undergo the addition of Si–H fragments. Because of the high activation barrier, the rubber mixtures obtained using this catalyst are able to remain unchanged for 6 weeks, and under the exposure of high-intensity light, curing takes 5 min [72]. The disadvantage of these complexes is the formation of dehydrogenative silylation products, as well as a mixture of α/β-products [74].

The use of [Pt(COD)Me_2_] as a hydrosilylation catalyst in a photoinitiated process has been described in [75]. It was suggested, that the reductive elimination of ethane with the formation of a coordinatively unsaturated platinum complex is a key stage of the activation process. However, the most notable are catalysts based on cyclopentadienyl complexes of platinum(IV)—(η^5^-cyclopentadienyl)trialkylplatinum(IV)—such as [CpPtMe_3_], whose photolysis in the presence of reactive silane HSiR_3_ gives a complex of composition [CpPtMe(SiR_3_)H] [76]. Such catalysts have found application in the production of impression materials, adhesives, adhesive liners, and seals [76], and it has also been proposed to use silicon-containing encapsulants in the production of LED [77]. The limiting factor in the use of such catalysts is their high toxicity; in order to reduce it, a cyclopentadienyl moiety is modified by non-polar groups such as CH_3_-, R_3_Si-, etc. Also, the amount of catalyst can be reduced by adding photoinitiators (α-diketones, α-ketoaldehydes, etc.,) [78]. From the industrial point of view, the process of photoactivated hydrosilylation is limited for large-scale production because of the high cost of ligands and the synthesis of the complexes.

Authors in [79] found that [(Me-Cp)Pt(Me)_3_] complex combined with a suitable PS such as naphthalene can activate the fast UV-curing of silicone rubber via hydrosilation at ambient temperature. The incorporation of naphthalene can increase the quantum efficiency of [(Me-Cp)PtMe_3_] by 24.8%, decrease activation energy from 35.6 to 30.9 kJ mol^−1^, and improve conversion from about 70 to 100%. The UV-activated hydrosilation by [(Me-Cp)Pt(Me)_3_]/naphthalene is more likely to follow the first-order reaction kinetics. Moreover, the UV-cured silicone rubbers catalyzed by [(Me-Cp)Pt(Me)_3_]/naphthalene exhibit superior mechanical strengths, storage modulus, and thermal stability to rubbers cured by [(Me-Cp)Pt(Me)_3_], due to the higher conversion and crosslinking density. On the other hand, UV-activated hydrosilation with [(Me-Cp)Pt(Me)_3_]/naphthalene shows the potential to fabricate complicated architectures by photolithography.

### 2.12. Hydrosilylation with Discrete Platinum Particles

Obtaining reduced discrete metal atoms that are stable in liquid solvents by in situ reduction of an ionic metal precursor is a big challenge. Authors in [80] showed that a liquid surfactant polydimethylsiloxane-polyethylene glycol (PDMS-PEG) (PEG = polyethylene glycol) enabled the synthesis of stable discrete platinum atoms (Pt1) by reducing Pt(IV) and Pt(II) salts. Authors reported the preparation of discrete mononuclear platinum atoms (Pt1) in a crown ether ([15]crown-5), as a structurally much simpler solvent, and the prepared [Pt1([15]crown-5)] was demonstrated for ultra-high catalytic activity and selectivity in hydrosilylation reactions. A combination of spectroscopic characterizations proves the reduced Pt species is Pt1(0) with a partially positive charge. 195Pt NMR and DFT calculation indicate the Pt1(0) is stabilized by the pseudo octahedral structure of [Pt1([15]crown-5)] involving two adjacent oxygens from the crown ether ring, although the oxygens in the crown ether ring have been known to host and stabilize certain metal cations. The [Pt1([15]crown-5)] shows ultrahigh activity (TOF of 8.3 × 108 h−1) with excellent terminal adducts selectivity in catalytic olefin hydrosilylation. This catalyst was found to be highly stable under hydrosilylation conditions. For example, the turnover number (TON) exceeded 1.0 × 109 for hydrosilylation between 1-octene and [(Me3SiO)2MeSiH] without showing signs of deactivation; the TON exceeded 2.0 × 108 while the catalyst remained active for a catalytically more demanding reaction between styrene and [(Me3SiO)2MeSiH].

### 2.13. Mechanistic Analysis of Hydroslylation Reaction

The first hydrosilylation reaction mechanism was proposed 50 years ago by Chalk and Harrod, and subsequently, it has been widely used for platinum catalysts [81]. The classical mechanism includes the following steps: I. oxidative addition of hydrosilane platinum; II. coordination of the olefin to the platinum center; III. migratory insertion of a coordinated olefin into Pt–H bond; IV. reductive elimination of the hydrosilylation product. It was suggested that steps I–III are reversible, while step IV was considered as irreversible and rate-determining. The formation of colloidal platinum(0) particles, which is observed during the hydrosilylation reaction, is associated with catalyst deactivation and leads to an increase in the contribution of undesired processes of the dehydrogenative silylation and the Si–Si combination in hydrosilanes. At the same time, a number of mechanistic studies were carried out for less active metals, the generalized mechanism was significantly different from the Chalk-Harrod one. Reactions in the presence of a Karstedt’s catalyst have the greatest demand for studying the mechanism. The main factor complicating the determination of intermediates is the low stability of the catalyst in a concentrated state, leading to the formation of colloidal platinum in the process of product isolation. Over the past two decades, a few papers with a detailed study of the mechanism of the hydrosilylation reaction catalyzed by platinum(0) complexes have been published [3,4]. The work [82] gives an example of studying the hydrosilylation reaction mechanism by nuclear magnetic resonance (NMR) and gas chromatography-mass spectrometry (GC-MS). By studying the dependence of conversion and reaction rate on alkene concentration, it was established that an increase in the concentration decreases the rate of the process.

**Figure 18:**
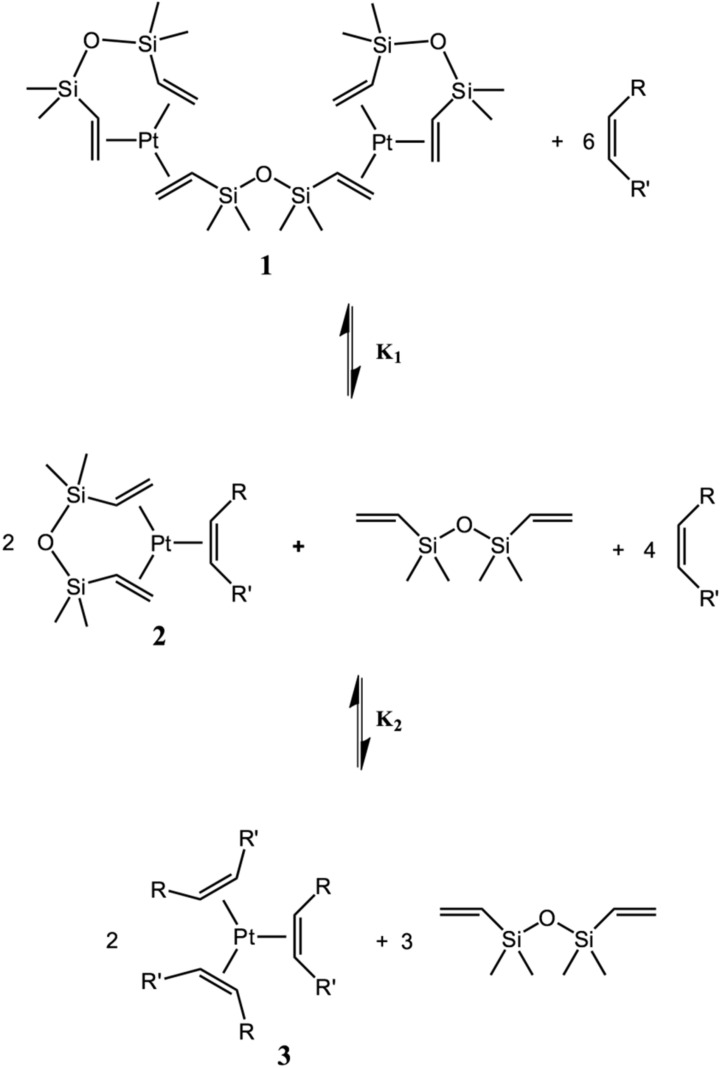
Equilibrium in the system “Karstedt’s catalyst—inhibitor.”.

Later it was experimentally established that the hydrosilylation reaction is negative (−1) order for alkene and first order for silane [20]. By studying the dependence of the reaction rate on the concentration of various silanes, it was found that an increase in the number of oxygen substituents at the silicon atom reduces the value of the rate constant. A linear correlation was observed between the chemical shift of the signal in the ^29^Si NMR spectrum of silane and the half-life of the deficient alkene. The formation of complexes, which are coordinated with alkenes, is reversible. Equilibrium is established between mono- and tricoordinated complexes (Figure 18).

**Figure 19:**
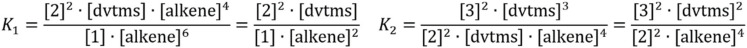
Equilibrium constants of the formation of complexes of platinum(0) with alkenes.

Using the equilibrium reaction (Figure 19) model for the formation of inhibited complexes, the equilibrium constants were expressed in terms of the concentration of intermediate platinum complexes 1–3 and alkene. The ratio of platinum complexes was determined by integrating peaks in ^195^Pt NMR spectra.

In the same work, it was shown that the introduction of norbornene instead of 1-octene leads to a significant increase in the equilibrium constant by a factor of 105, which indicates the introduction of ligands with sterically hindered substituents. However, some alkenes are practically not capable to replace divinylsiloxane ligands in terms of stability of complexes. Also, during the catalytic process, a reversible isomerization of alkene is possible, which was established by deuterium-labeling experiments [21]. 

The mechanism of the hydrosilylation reaction was studied by calculation methods using the density functional theory (DFT) in [83,84]. In this work, for the first time, the possibility of isomerization of the main product via Berry’s mechanism (pseudo-rotation of coordinated to platinum ligands) is proposed.

The formation of platinum nanoparticles during the hydrosilylation process was established by transmission electron microscopy and dynamic light scattering. The use of dibenzocyclooctatetraene for inhibition of a homogeneously catalyzed hydrosilylation reaction showed that this process can take place at the colloidal platinum[85]. In [86], it was shown that the use of platinum nanoparticles with an average size of 2 nm provides the efficiency of the hydrosilylation of 1-octene by polymethylhydrosiloxane, which is comparable to that for Karstedt’s catalyst.

## 3. Conclusions

The hydrosilylation reaction holds a special place among the most important industrial applications of homogeneous catalysis, making it possible to obtain multifunctional silanes and silicone materials for the production of adhesives, casting compounds, and polymers [81]. The reaction is characterized by a high yield, a wide range of substrates, and resistance to oxidation by atmospheric oxygen. Because of high reproducibility and selectivity, currently, catalysts based on platinum group metals are used to obtain most of the above listed products. The Karstedt’s catalyst, being the most versatile with the relative simplicity of its preparation, is the most commonly used in the silicone industry. However, the disadvantages of this catalyst are low stability at elevated temperatures, the tendency to be completely deactivated by the sulfur-containing compounds, low reproducibility, and excessively high activity at room temperature. Despite the fact that the reaction has been used for 50 years, the mechanism, as well as the nature of the intermediates, is still poorly understood. The reason for this is the short lifetime of the intermediates as well as the limited capabilities of previous spectral methods.

In conclusion, the hydrosilylation of olefins is one of the key reactions to obtain industrially important organosilicon derivatives, such as organofunctional silanes and silicones. Applied research in this area made an important contribution to the development of new hydrosilylation catalysts, as well as new methods for improving the selectivity, stability, and activity of the developed catalysts. Over the past decade, a number of methods and approaches have been proposed, such as the development of heterogeneous hydrosilylation catalysts [64], as well as the use of platinum nanoclusters [87].

However, the lack of a rational explanation of the numerous experimental data, as well as patterns in the results of the hydrosilylation mechanism investigation, demonstrates that a deeper study of the principles of action of inhibitors and promoters is necessary. Often the results of various studies of the mechanism are difficult to compare with each other because of different conditions (heterogeneous/homogeneous catalysis, solvents, etc.,). In turn, catalytic systems for industrial processes must be developed individually according to the requirements for raw materials and the final product. For example, the industrial production of organofunctional silanes requires high selectivity of the catalyst used, while the curing of silicone rubbers requires their high activity and low cost.

In almost all industrial hydrosilylation processes, catalysts based on platinum compounds are used. The main disadvantage of these processes is the impossibility to reuse platinum. In general, there are three main types of industrial hydrosilylation catalysts:

(1) Most of these catalysts are used in the production of silicone coatings and self-sealing tapes. This application requires an increased rate of coating formation, which at the moment is provided by a relatively high concentration of catalyst (100–200 ppm). In this regard, the development of available and cheap catalysts possessing high activity is relevant.
(2) In the production of silicon rubber mixtures, the determining factor is a long shelf life, as well as high conversion. Therefore, for the silicone rubber industry, the catalysts that exhibit temperature-controlled activity and provide increased TOF values are required. A decrease in platinum concentration due to increased catalyst stability is also encouraged.
(3) An equally important area is the preparation of organosilanes with various functional groups. In this case, the hydrosilylation reaction is crucial. Catalysts with high selectivity for a particular Si–H bond addition product and with increased stability in the catalytic cycle are the most demanded.
